# Comparing the Effects of Intramuscular Injections of Dexamethasone and Ketorolac Tromethamine on Post-treatment Endodontic Pain: An In Vivo Study

**DOI:** 10.7759/cureus.57086

**Published:** 2024-03-27

**Authors:** Reshma M Sekhar, Sravani Nirmala, Rakesh Reddy Chukka, Srikanth Goud G, Naresh Kumar K, Deepu Patil

**Affiliations:** 1 Conservative Dentistry and Endodontics, Dr. Syamala Reddy Dental College Hospital and Research Centre, Bengaluru, IND; 2 Conservative Dentistry and Endodontics, SVS (Sri Venkata Sai) Institute of Dental Sciences, Mahbubnagar, IND; 3 Oral Medicine and Radiology, SVS (Sri Venkata Sai) Institute of Dental Sciences, Mahbubnagar, IND; 4 Conservative Dentistry and Endodontics, AME's (Academy of Medical Education's) Dental College and Hospital, Raichur, IND

**Keywords:** nsaid, verbal rating scale, debridement, symptomatic irreversible pulpitis, analgesic, anesthetic, corticosteroids, single visit root canal therapy, post endodontic pain, steroid

## Abstract

Background and objectives: Pain is the primary reason dental patients seek endodontic therapy. Post-treatment endodontic discomfort is a sequelae of periapical inflammation and anti-inflammatory drugs such as corticosteroids and non-steroidal anti-inflammatory drugs (NSAIDs) would be reasonable therapy options. The purpose of this study was to compare and assess the efficacy of intramuscular injections of dexamethasone and ketorolac tromethamine versus placebo in reducing post-treatment endodontic pain in individuals undergoing root canal treatment.

Methodology: Patients diagnosed with symptomatic irreversible pulpitis were selected. Nonsurgical endodontic therapy was carried out in a single visit. After completion of the root canal therapy, the patients were randomly assigned to one of the three groups for intramuscular drug administration. In group 1, 2 ml of sterile saline was administered, in group 2, 1 ml of 4 mg dexamethasone was administered; and in group 3, 1 ml of 30 mg ketorolac tromethamine was administered. Preoperative and postoperative pain intensity was measured by a verbal rating scale. Postoperatively, the incidence and severity of pain were recorded after four, 24, and 48 hours.

Results: All three groups showed a highly statistically significant reduction in pain scores when compared to preoperative levels. At the end of four hours, dexamethasone and ketorolac tromethamine showed highly significant results. Dexamethasone significantly reduced pain after 24 hours when compared to ketorolac and placebo groups. At the conclusion of 48 hours, all three groups experienced a gradual decrease in pain levels.

Conclusion: Effective and complete debridement of infected root canal system provides predictable gradual reduction of post-endodontic pain.

## Introduction

Although dentists are capable of controlling pain at the time of treatment with a variety of anesthetic, analgesic, and sedation techniques, the management of the patient’s post-treatment pain remains a significant problem [1]. According to the literature, the frequency of post-treatment endodontic pain ranges from 1.5% to 53% [2]. It has been stated that periapical tissue violation is a major contributing cause of post-treatment pain. The noxious agents may include apical extrusion of debris or irritants, incomplete instrumentation that results in changes in the endodontic microbiota or environmental conditions, and secondary intraradicular infections that are induced or exacerbated during root canal treatment, causing tissue injury and inflammation [3,4].

Inflammation has been defined by Robbins and Cotran as "the local reaction of vascularized tissue to injury." Chemical mediators are released due to tissue injury which further induces vascular changes. As a result, vascular permeability and fluid flow are increased with resultant increased pressure at the site of inflammation. As etiological factors often cannot be precisely determined, a rational treatment would include intracanal medicaments or systemic medications such as narcotic analgesics, steroids, or non-steroidal anti-inflammatory drugs (NSAIDs) [5]. 

Corticosteroids potentially will ward off endodontic pain due to suppression of the release of inflammatory mediators at the site of injury. They can be used both locally and systemically in the prevention and treatment of endodontic diseases. Dexamethasone is a synthetic adrenocorticosteroid. When compared to other steroids, it is potent and relatively safe with no sodium retaining potential. It has 20-fold greater anti-inflammatory activity compared to hydrocortisone [6].

NSAIDs have been shown to be extremely effective in the treatment of postoperative surgical pain and other types of pain reduction from a variety of etiologies such as oral surgery, cancer, migraine, and odontalgia. Ketorolac tromethamine is a new non-opioid analgesic, an NSAID. It is a member of pyrrolo-pyrrole group, and its primary mode of action is the inhibition of the production of prostaglandins through the inhibition of the cyclooxygenase pathway that metabolizes arachidonic acid [7-8].

Direct comparisons of pretreatment, treatment, and post-treatment pain are extremely rare. There are very few studies available in the literature on the use and comparison of these agents to reduce post-treatment endodontic pain. Hence this study compares dexamethasone and ketorolac tromethamine injections with placebo in reducing post-treatment endodontic pain using a double-blind design on patients undergoing root canal therapy.

## Materials and methods

This was a randomized, double-blind clinical trial conducted at the Academy of Medical Education's (AME's) Dental College & Hospital, Raichur, Karnataka, India. AME Ethical Committee, Raichur, approved the study (approval number: AME/DC/CONS2013).

Patient selection

Ninety male patients in the age group of 20-50 years visiting AME’s Dental College & Hospital, who were diagnosed with symptomatic irreversible pulpitis and indicated for non-surgical root canal therapy of any molar teeth, were included. The patients' demographic details and a thorough history of the present illness were recorded. Clinical and radiographic evaluations were performed and recorded. Patients with systemic conditions for whom steroids and NSAIDs should be cautiously used were excluded. Patients who fulfilled the inclusion criteria were selected after signing the informed consent.

Treatment procedure

The patient’s age, tooth number, degree of pain, and sensitivity to percussion (yes or no) were recorded. Isolation was obtained with a rubber dam application after successful anesthesia with 2% lidocaine. Single-visit non-surgical endodontic therapy was performed by an endodontist. The access cavity was prepared, followed by the determination of the working length using an apex locator (J. Morita Corporation, Saitama, Japan) and a radiograph (digital RVG; Carestream Dental LLC, Atlanta, Georgia, United States). Biomechanical preparation was carried out using the crown-down pressure-less technique using K3XF rotary files. Copious irrigation with 3% sodium hypochlorite and saline was accomplished using a 27-gauge side-vented needle. Obturation was accomplished using lateral compaction of gutta-percha with AH Plus sealer (Dentsply Sirona, Charlotte, North Carolina, United States), followed by post-endodontic restoration with glass ionomer cement (GIC). The tooth was relieved of occlusion at the end of the procedure.

An intramuscular injection of 2 mL of solution was given at the site of the deltoid muscle using a 25-gauge, 16-mm needle. Administration was performed in a double-blind fashion. The three solutions were coded by an uninvolved party unknown to the operator or patient and the code was not broken until the completion of the study.

Drugs and methods of administration

After completion of the root canal therapy, the patients were randomly assigned into three groups with 30 patients in each. The drugs/distilled water (placebo) given to each group is shown in Table 1.

**Table 1:** Groups and the drugs used for intramuscular administration

Groups	Material	Number of patients
Group-I	Distilled water (2 ml)	30
Group-II	Dexamethasone 2 ml (4 mg/2 ml)	30
Group-III	Ketorolac tromethamine 1 ml (30 mg/ml)	30

Data collection

Pretreatment and post-treatment pain intensity was recorded using the Verbal Rating Scale (VRS). Data collection on the incidence and severity of post-treatment pain was recorded at four, 24, and 48 hours. Scores of 1, 2, 3, or 4 were given. The criteria used for scoring are given in Table 2.

**Table 2:** Pain intensity scoring criteria

Score	Description of pain
1	None
2	Mild, requiring no analgesics
3	Moderate, relieved by analgesics
4	Severe, unrelieved by analgesics

Post-treatment pain incidence and severity were recorded by the operator by calling the patient on phone after four, 24, and 48 hours. Patients were prescribed a reserve medication of oral ibuprofen (400 mg) and instructed to take the medication only if pain persists and is intolerable.

Statistical analysis

Data was entered in an MS Excel sheet (Microsoft Corporation, Redmond, Washington, United States) and subjected to statistical analysis using IBM SPSS Statistics for Windows, Version 19.0 (Released 2010; IBM Corp., Armonk, New York, United States). All values were presented in terms of the mean and SD. The normality of the data was tested using the Kolmogorov and Smirnov methods. Comparisons between the two groups were done using the unpaired t-test for parametric data and the Mann-Whitney U test for non-parametric data. Comparison of post-treatment pain scores between time intervals within each group was done by using the Friedman test followed by the posthoc Dunn's multiple comparisons test, and comparison of post-treatment pain scores between groups at different time intervals was done by using the Kruskal-Wallis test followed by the posthoc Dunn's multiple comparisons test. A p-value less than 0.05 was considered statistically significant.

## Results

**Figure 1:**
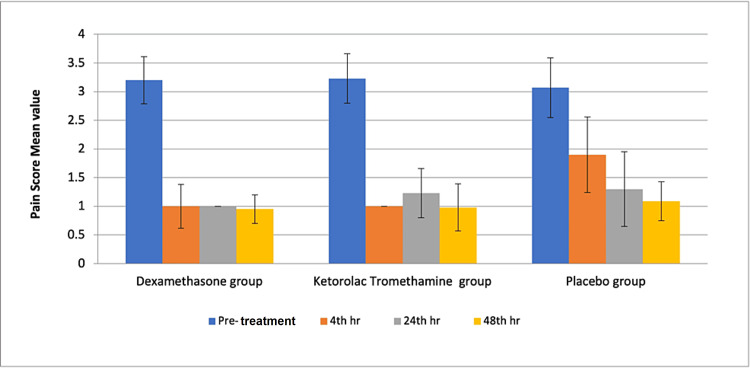
Comparison of post-treatment pain score between groups at different time intervals

The reduction of post-treatment pain was highly significant with dexamethasone and ketorolac when compared to placebo at the fourth hour. At 24 hours, dexamethasone showed a significant reduction in post-treatment pain, and at 48 hours, post-treatment pain between all the groups was not statistically significant. This showed that dexamethasone was superior in relieving post-endodontic pain when compared with ketorolac, tromethamine, and placebo (Figure 1).

The results of the study show the ability of dexamethasone and ketorolac tromethamine to reduce post-treatment pain (P<0.05) as compared to the control (placebo). in all three groups, both maxillary and mandibular teeth were included.

## Discussion

Post-treatment pain is defined as pain of any degree that occurs after the initiation of root canal therapy and develops when the integrity of the periapical tissues is compromised [9]. This can occur during endodontic treatment from mechanical irritants such as hand instruments and obturation materials protruding beyond the minor foramen. Chemical irritation can occur if any of the solution is extruded beyond the apex. Sealers used in obturation are often both mechanical and chemical irritants, as many commercially available sealers are cytotoxic [10].

An inflammatory response is initiated in response to tissue injury, which leads to an influx of inflammatory cells and mediators, resulting in the activation and sensitization of nociceptors, leading to peripheral and central sensitization, and resulting in post-treatment pain. Transmission of pain signals initiated by tissue damage leads to sensitization of peripheral and central pain pathways. Many inflammatory mediators function to change the transduction sensitivity of peripheral neurons. Prostaglandins cause vasodilation in local blood vessels, chemotaxis of inflammatory cells, and sensitization of the receptors of pain fibers by stimulating other mediators. Leukotrienes affect vascular permeability and increase pain through prolonged stimulation of nerve fibers. Substance-P (SP) and calcitonin gene-related peptide (CGRP) are the neuropeptides released by C-fibres upon stimulation, causing vasodilation, increased vascular permeability, and pain by lowering sensory nerve thresholds. Accumulation of these mediators in the dental pulp leads to increased vascular permeability, pain, and tissue necrosis [11].

In the present study, 90 patients with symptomatic irreversible pulpitis had mean pretreatment baseline pain scores as moderate and severe. In this study, the age categories were seen to be evenly distributed among the groups. Age seems to have only a mild influence on the experience of post-endodontic pain. On analyzing the distribution of types of teeth, both maxillary and mandibular teeth seem to be equally divided among the groups. A greater incidence of pain is usually seen in the posterior teeth compared to the anterior teeth, and the mandibular compared to the maxillary [12]. This could be attributed to the greater number of root canals and foci of pain in posterior teeth. Hence, in the present trial, only posterior teeth were included to reduce the potential bias due to tooth type.

A rubber dam was used in all cases for control of cross-infection, protection, and improving treatment efficacy, which allowed the use of desired irrigants. In the present study, cleaning and shaping were carried out by the crown-down pressure-less technique with K3XF rotary files, which flared the canals from coronal to apical, resulting in a potential decrease in hydrostatic pressure apically and minimizing the passage of materials and tissue debris into the periapical tissues, thus preventing post-endodontic pain or flare-ups. In the study by Goreva et al., results showed that constantly tapered K3 instrumentation showed more dentine removal towards the outer aspect of the curve, while progressively tapered ProTaper files (Dentsply Sirona) tended to transport towards the furcation coronally [13]. Constantly tapered K3 instrumentation shows more dentine removal towards the outer aspect of the curve, while progressively tapered ProTaper files tend to transport towards the furcation coronally [14].

Standardized gutta percha with AH Plus sealer was used as the core obturation material in all cases, as it has been considered the most adaptable core material for endodontic filling. Obturation was done on the same visit, particularly when treating cases with irreversible pulpitis. Single-visit endodontic treatments have become more common due to advancements in dental materials, such as nickel-titanium rotary files. Studies show similar healing rates and lower post-operative pain in single-visit treatment groups. Endodontic treatment with a single visit has better antimicrobial efficacy in apical periodontitis [15]. In a study by Kvist et al., the antimicrobial efficacy of endodontic treatment completed in one visit was evaluated as compared to two-visit treatment in teeth with apical periodontitis [16]. Microbial samples retrieved were 29% of the one-visit group and 36% of the two-visit group.

Occlusal reduction was performed in the same visit as it helps in reducing post-treatment pain. In a study by Rosenberg et al., it was concluded that occlusal reduction prevents postoperative pain in those patients whose teeth initially exhibit pulp vitality, percussion sensitivity, preoperative pain, and/or the absence of peri-radicular radiolucency [17].

The main objective of the present study was to evaluate and compare the effect of intramuscular injections of dexamethasone (4 mg/2 ml) and ketorolac tromethamine (30 mg/1 ml) on post-endodontic pain in patients with symptomatic irreversible pulpitis undergoing single-visit endodontic therapy using a VRS.

Dexamethasone is a synthetic adrenocorticosteroid that is used to treat a variety of inflammatory and auto-immune conditions. Corticosteroids can prevent or suppress inflammation in response to multiple inciting events, including radiant, mechanical, chemical, infection, and immunological stimuli. Corticosteroids interact with specific receptor proteins in target tissues and regulate the expression of corticosteroid-responsive genes, thereby changing the levels and array of proteins synthesized. Lipocortin, a steroid-induced protein, has antiphospholipase A2 activity, inhibits arachidonic acid synthesis, and thereby reduces cyclooxygenase and lipoxygenase products. A reduction in pulpal levels of both PGE2 and IL in cases of untreated irreversible pulpitis has been demonstrated by intraosseous injections of glucocorticoids [18].

When compared to NSAIDs, glucocorticoids can reduce bradykinin levels and can even suppress edema with the release of vascortin protein. All NSAIDS have greater potency as analgesics and antipyretics than as anti-inflammatory agents [19]. The plasma half-life of dexamethansone is 200 minutes, and the tissue half-life is 75 hours. Its peak anti-inflammatory activity is seen at around 12-24 hours. The maximum recommended dose is 0.75-9 mg per day in divided doses every 6-12 hours [20].

Ketorolac tromethamine is an NSAID that is a potent analgesic but only moderately effective anti-inflammatory. It is a member of the pyrrolo-pyrrole group of NSAIDs. NSAIDs' analgesic effects are attributed to inhibiting prostaglandin synthesis in the periphery, preventing peripheral sensitization. Ketorolac irreversibly acetylates the COX enzyme, blocking prostaglandin production, which sensitizes nerves and contributes to peripheral sensitization. It is introduced as an injectable drug and is effective for the treatment of endodontic pain. Its plasma half-life is four to six hours, with a peak plasma concentration of 60-90 minutes. Its analgesia peaks at two to three hours and lasts for six hours. The recommended dosage for a day is 40 mg/ml [20-21]. In this study, a dose of 30 mg was given, although a range of 10-40 mg has been administered for endodontic pain in previous studies [20].

The mean pain intensity (VRS) was either moderate or severe at baseline for all three groups (Table 2). Various studies have reported the association between pretreatment and post-treatment pain [22]. In our study too, there was a strong correlation between pretreatment and post-treatment pain among all three groups. The mean pain intensity postoperatively decreased in all three groups at the fourth, 24th, and 48th hours. All three groups showed a statistically highly significant (p<0.0001) reduction in pain scores when compared to pretreatment scores.

At the end of four hours, dexamethasone showed highly significant results (p<0.0001) when compared with thesixplacebo. This can be attributed to the fact that steroids have a greater anti-inflammatory action and may lead to greater analgesic properties in post-endodontic pain relief [19]. When compared to placebo, ketorolac showed a highly significant (p<0.0001) reduction in pain scores due to its potent analgesic effect, which lasts for 6 hours [21]. Post-endodontic pain scores compared between the dexamethasone and ketorolac tromethamine groups were not significant (p > 0.05). This may be attributed to the fact that, due to the greater anti-inflammatory potency of corticosteroids and the peak analgesia of ketorolac tromethamine, both groups showed no pain. Forbes et al. showed that ketorolac was significantly superior to aspirin for every measure of total and peak analgesia and significantly superior to acetaminophen-codeine for measures of total effect [22]. The analgesic effect of ketorolac in their study was significant after one hour and persisted for six hours. In the study by Abbas et al., it was concluded that 10 mg of ketorolac affords better pain relief with fewer side effects than hydrocodone/acetaminophen [23]. Placebo also showed a reduction in pain post treatment, but it was less compared to the dexamethasone and ketorolac groups and was not statistically significant. This could be explained as even though the complete pulp was removed in placebo groups, inflammation and its mediators played an important role in endodontic pain as no drugs were used.

At the end of 24 hours, dexamethasone showed a statistically significant (p<0.05) reduction in pain compared to the ketorolac and placebo groups. This can be attributed to its greater potency and long-lasting anti-inflammatory effects, as it has a tissue half-life of 75 hours. An increase in pain scores was observed in the ketorolac group (p = 0.95), which can be attributed to its plasma half-life of around six hours, after which inflammation would have increased [22]. Steroids have greater anti-inflammatory action and possibly lead to greater analgesic properties than NSAIDS in pain conditions, where multiple inflammatory mediators are present and contribute to the development of inflammation and pain [19].

Post-endodontic injection is expected to have an extended duration of action for the analgesic effect due to the prevention of peripheral sensitization. This can be attributed to the insufficient anti-inflammatory potency of ketorolac, which is a more potent analgesic than an anti-inflammatory agent. This can also be due to the inflammatory state of the tissues after the insults during the treatment procedure. In a study by Siqueira and Barnett, ketorolac worked quite well for most of the patients; almost 77% had no pain, and only 23% had mild pain. The development of pain and its severity are more dependent on the intensity of tissue damage due to mechanical, chemical, and/or microbial injuries and their persistence. Even with the utmost care taken during cleaning and shaping procedures (use of the apex locator crown-down pressure-less technique), some amount of extrusion is unavoidable [10].

In the study by Marshall and Liesinger, at the end of 48 hours, there was a reduction in pain scores (p = 0.32) in the dexamethasone, ketorolac, tromethamine, and placebo groups, suggesting a gradual reduction in inflammatory pain [24]. This is similar to Harrison's study, where over 90% of patients reported no or mild pain after 48 hours of obturation, and further reduction in tissue inflammatory reactions [25]. However, in the current study, the pain reduction scores between dexamethasone and placebo (p = 0.65) were not significant. Pain reduction scores between ketorolac tromethamine and placebo (p = 0.65) were not significant. The pain reduction scores between dexamethasone and ketorolac tromethamine (p = 0.36) were also not significant.

This gradual reduction in inflammation was due to the host response. Macrophages, known to mediate inflammation, influence healing in a positive way by increasing angiogenesis, decreasing bacterial loads, phagocytosing debris, and providing matrix deposition [25]. Reduction of pain is based on reducing tissue levels of inflammatory mediators and elevated interstitial tissue pressure that stimulates peripheral terminals of nociceptors [26].

There are a few limitations of the present study. Only male patients were included in the study to eliminate bias as the pain threshold is lower in females when compared with males. Mandibular posterior teeth were chosen for the present study because a greater incidence of pain is usually seen in posterior teeth compared to anterior teeth and mandibular compared to maxillary. This could be attributed to the greater number of root canals and foci of pain in posterior teeth. Patients with periapical lesions or radiolucency were excluded from the study, as it was controversial if periapical lesion size or radiolucency would influence postoperative pain [12]. There was a decrease in pain after medication, which cannot be evaluated clearly from follow-up in 24-48 hours

Dexamethasone administered intramuscularly is clinically successful and avoids the need for repeated post-treatment doses; nevertheless, operator experience, patient pain, and the addition of additional equipment may be limiting considerations [24]. As the frequency and severity of side effects may increase with prolonged use of injectable ketorolac, its usage should be restricted to short-term therapies (not exceeding five days) [8].

## Conclusions

By the fourth hour, dexamethasone and ketorolac significantly reduced post-treatment discomfort compared to placebo. When it came to post-treatment pain, dexamethasone demonstrated a statistically significant reduction after 24 hours, whereas at 48 hours, there was no statistically significant difference in pain between any of the groups. This demonstrated that, in comparison to ketorolac, tromethamine, and placebo, dexamethasone was more effective in reducing post-endodontic pain.

Endodontic pain is more of an inflammatory pain than a neurogenic pain. Thus, thorough debridement of the infected pulp tissue, cleaning and shaping of the root canal with minimal instrumentation without exceeding the apical foramen, and filling with a hermetic seal prevent and minimize the chances of inflammation and reinfection. However, mild inflammation or post-treatment endodontic pain, which is unavoidable, will be taken care of by the host response without any requirement for steroids or NSAIDs.
